# Parliament and the Profession

**Published:** 1918-11-09

**Authors:** 


### Parliament and the Profession.

### The Influenza Epidemic.

Several points were brought out in the replies of Ministers to questions with regard to the epidemic of influenza. The President of the Local Government Board did not consider that the outbreaks of influenza and septic pneumonia could be considered as two separate epidemics, and stated that the rise in mortality is not so great as that experienced in Paris and Vienna. Numerous conferences had taken place between public departments and bacteriological investigations had been carried on continuously since the commencement of the summer outbreak which showed that this epidemic does not differ from other outbreaks, the fatality being due to secondary infections, chiefly by pneumococci and streptococci. The Minister of National Service assured a questioner that withdrawals of doctors from civil life had been made with due regard to the contingency of a widespread epidemic disease affecting the population. Sir Arthur Geddes added that concurrently there had been increased need for medical officers at the Fronts, and the examination of men of military age employed many other medical men. A very large number of doctors had been released from the call-up in order that they might devote their whole time to civil practice. A number of naval surgeons are assisting with the armies in France.

The Chief Secretary for Ireland stated that there had been 118 cases of influenza amongst prisoners in Belfast Gaol.

### War Bonuses and Medical Benefit.

An answer given by Sir Edwin Cornwall to Mr. Currie indicated that the position of a non-manual worker, receiving in wage and bonus over £160 a year, would be that he would be disentitled to medical benefit under the National Health Insurance Act.

### Major Chapple and Army Nurses.

Major Chapple asked the Under-Secretary of State for War whether his attention has been called to the fact that the London Hospital nurses only get two years' training for their certificate; and whether any such nurses have recently been appointed to the Army nursing staff; and whether he is aware that recently qualified nurses appointed to the Army nursing staff from all the chief Scottish hospitals hold certificates showing four years' training, that similar nurses appointed from the great Metropolitan hospitals hold certificates showing three years' training, and that nurses appointed from the London Hospital hold certificates showing only two years' training; and would he explain why this standard of training is accepted from the London Hospital. Mr. Macpherson stated that no nurse from the London Hospital with less than three years' training and service in the wards of the hospital is appointed to the Queen Alexandra's Imperial Military Nursing Service.

### Women's Occupation and its Effects.

Sir George Cave stated that the Factory Department of the Home Office has been making careful inquiry into the effects of the employment of women in new processes during the war, particularly with regard to the suitability of the work for women from the point of view of health. A report on this subject is nearly ready, and will in due course be published.

### Consumption of Alcohol in Military Hospitals.

Mr. Chancellor asked Mr. Macpherson what was the consumption of malt liquors and of wines and spirits by officers and other ranks in a given period in military, war, Territorial general hospitals, and hospitals established by the War Office in Poor-Law institutions in the London District. Mr. Macpherson said that to obtain these facts would throw a great deal of labour upon the staffs of the hospitals, particularly now when they are overworked.

### London Hospital.

Major Chapple has given notice of the following motion: "That the system carried on at the London Hospital under which nurses are taken from their training in the wards at the end of their second year, sent out to attend private cases, paid at the rate of 13s. 5d. per week while they receive £2 12s. 6d., the hospital appropriating the difference of 39s. per week earned by them, is adopted by no other great hospital in Britain, gravely interferes with the professional training in the wards of such nurses during their third and most important year, and is a cruel exploitation of women for the sake of pecuniary gain, and this House calls upon the Government to introduce legislation to remedy the abuse."

